# Comparing Clinical Perimetry and Population Receptive Field Measures in Patients with Choroideremia

**DOI:** 10.1167/iovs.18-23929

**Published:** 2018-07

**Authors:** Edward H. Silson, Tomas S. Aleman, Aimee Willett, Leona W. Serrano, Denise J. Pearson, Andreas M. Rauschecker, Albert M. Maguire, Chris I. Baker, Jean Bennett, Manzar Ashtari

**Affiliations:** 1 Section on Learning and Plasticity, Laboratory of Brain and Cognition, National Institute of Mental Health, National Institutes of Health, Bethesda, Maryland, United States; 2 Center for Advanced Retinal and Ocular Therapeutics (CAROT), University of Pennsylvania, Philadelphia, Pennsylvania, United States; 3 F.M. Kirby Center for Molecular Ophthalmology, Scheie Eye Institute, University of Pennsylvania, Philadelphia, Pennsylvania, United States; 4 Department of Radiology, University of Pennsylvania, Philadelphia, Pennsylvania, United States

**Keywords:** choroideremia, retinotopy, fMRI, population receptive fields

## Abstract

**Purpose:**

Choroideremia (CHM) is an X-linked recessive form of hereditary retinal degeneration, which, at advanced stages, leaves only small central islands of preserved retinal tissue. Unlike many other retinal diseases, the spared tissue in CHM supports excellent central vision and stable fixation. Such spared topography in CHM presents an ideal platform to explore the relationship between preserved central retinal structure and the retinotopic organization of visual cortex by using functional magnetic resonance imaging (fMRI).

**Methods:**

fMRI was conducted in four participants with CHM and four healthy control participants while they viewed drifting contrast pattern stimuli monocularly. A single ∼3-minute fMRI run was collected for each eye separately. fMRI data were analyzed using the population receptive field (pRF) modeling approach. Participants also underwent ophthalmic evaluations of visual acuity and static automatic perimetry.

**Results:**

The spatial distribution and strength of pRF estimates correlated positively and significantly with clinical outcome measures in most participants with CHM. Importantly, the positive relationship between clinical and pRF measurements increased with increasing disease progression. A less consistent relationship was observed for control participants.

**Conclusions:**

Although reflecting only a small sample size, clinical evaluations of visual function in participants with CHM were well characterized by the spatial distribution and strength of pRF estimates by using a single ∼3-minute fMRI experiment. fMRI data analyzed with pRF modeling may be an efficient and objective outcome measure to complement current ophthalmic evaluations. Specifically, pRF modeling may be a feasible approach for evaluating the impact of interventions to restore visual function.

Retinotopic mapping with functional magnetic resonance imaging (fMRI) has been widely used to reveal the systematic representation of visual space in the cerebral cortex (for review see Ref. 1). During these scans, participants typically fixate on the center of a screen while a visual stimulus (e.g., high-contrast checkerboard) moves systematically through the visual field, allowing for estimation of the preferred retinotopic location of each voxel.^2–4^ Population receptive field (pRF) modeling,^5–7^ an advanced method as compared to standard retinotopic mapping paradigms, uses a computational model of visual responses to provide an objective and efficient estimate of the visual space over which the activity of each voxel can be modulated.^8^ Although standard retinotopic mapping and pRF modeling have been used primarily to study the visual system of healthy volunteers with no history of ocular disease, a number of studies have applied these techniques to patients with ocular disease, primarily age-related macular degeneration,^9–11^ but also glaucoma,^12^ retinitis pigmentosa,^13^ and patients with homonymous visual field deficits.^14^ In this study, we applied pRF modeling to patients with choroideremia (CHM).

CHM is an X-linked inherited form of retinal degeneration.^15–21^ Patients typically present in the second decade of life with nyctalopia (night blindness), followed by progressive constriction of the visual field, while their central vision is spared even into the sixth decade of life.^22–29^ This relatively preserved central vision is subserved by islands of retinal tissue that are separated from the surrounding blind retina by steep transitions in the retinal structure, from a near normal appearance to complete atrophy.^22–29^ The “geographic” topography of the retinal structure and associated vision in this disorder offer a platform to explore the precise relationships between the remaining central and peripheral vision and the cortical representations of the visual field measured through fMRI.

The goal of this study was to assess the relationship between the visual functions measured through standard static automatic perimetry (SAP) and pRF estimates measured with fMRI in a group of participants with CHM at different disease stages.

## Methods

### Subjects

Four patients with molecularly confirmed CHM (Table 1) and four healthy control (HC) participants with normal ophthalmic examinations were included in this study (for full patient description see Supplementary Material and Supplementary Fig. S1). All participants underwent a complete ophthalmic examination and had their visual sensitivity measured with SAP. Informed consent was obtained from individual participants after explanation of the study procedures in compliance with the Declaration of Helsinki. The study consent form was approved both by the Children's Hospital of Philadelphia and the University of Pennsylvania Internal Review Board.

**Table 1:**
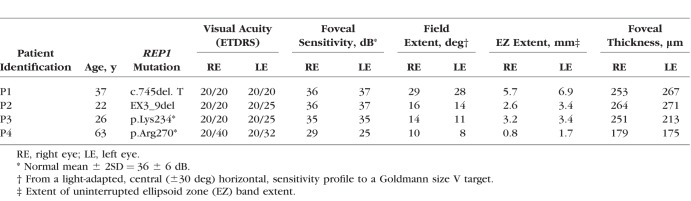
Clinical and Molecular Characteristic of Patients

### Retina Functional and Structural Measures

Achromatic, light-adapted SAP was performed with a modified system Humphrey Field Analyzer (HFA II-i; Carl Zeiss Meditec, Dublin, CA, USA) by using a conventional 10-2 testing protocol grid (200-ms duration, 0.45° diameter, achromatic stimuli) and a horizontal sensitivity profile that extended to 10° from fixation (200-ms duration, 1.7° diameter stimuli) in light- (achromatic stimuli) and dark-adapted (500-nm and 650-nm stimuli) conditions.^22,26,30^ The lateral extent of the central visual field was estimated by determining the eccentricity at which cone-mediated sensitivities within this profile were reduced to <5dB. Retinal imaging was performed with a spectral-domain optical coherence tomography (SD-OCT; Spectralis; Heidelberg Engineering, Carlsbad, CA, USA) system. SD-OCT was performed with 9-mm-long horizontal sections crossing the anatomical fovea.^22^

### fMRI Parameters

fMRI scans were conducted at the Children's Hospital of Philadelphia on a research-dedicated 3.0T Siemens Verio system (Erlangen, Germany) using a 12-channel head coil. All scans were performed by a single operator and monitored to be free of artifacts at the time of acquisition. PRF experiments were performed using a blood-oxygenation-level-dependent (BOLD) sequence, acquiring 100 volumes with 29 slices at 2.8 × 2.8 × 2.5-mm resolution (matrix, 64 × 64; repetition time [TR]/echo time [TE], 2000/30 ms; flip angle, 90°; field of view, 180 mm^2^ with total acquisition time of 3:24 minutes) in oblique orientation perpendicular to calcarine sulcus. Four brain volumes were acquired at the beginning of each fMRI experiment to allow reaching T1 equilibrium and were not used in the final analysis. pRF acquisitions were accomplished using a real-time fMRI function to monitor subjects' motion in real time. Scans were terminated if a subject's motion in three translational and rotational directions exceeded 1 mm or 1 degree, respectively.^31^

### Anatomic Imaging Parameters

A 3-D magnetization prepared rapid acquisition gradient echo (MPRAGE) sequence was used to obtain high-resolution T1-weighted anatomic scans with the following characteristics: TR/TE, 2080/2.43 ms; bandwidth, 180 Hz/Px; matrix size, 256 × 256; field of view, 256 × 256 mm^2^; 192 axial slices; slice thickness, 1 mm; inversion time, 1200 ms, with flip angle, 9°; number of excitations, 1; echo spacing, 7.3; integrated parallel imaging techniques, 2; and scan time, 5.45 minutes.

### pRF Mapping Stimulus

The pRF stimulus consisted of a bar within a circular aperture with 100% contrast drifting checkerboards traversing through the visual field. The bar stimuli made a total of 8 sweeps, with 12 evenly-spaced steps per sweep (1 step/TR). Specifically, the order of eight sweeps for each run were as follows: (1) left – right, (2) bottom right – top left, (3) top – bottom, (4) bottom left – top right, (5) right – left, (6) top left – bottom right, (7) bottom – top, and (8) top right – bottom left. The last six bar positions on each of the four diagonal sweeps were occluded to allow for baseline estimation.^7^ Bar stimuli were contained within a circular aperture with a total diameter of 22.5°. Each participant completed two runs with either the left eye or right eye occluded. Occlusion was controlled electronically via an MRI-compatible goggle system, which allowed stimuli to be presented to each eye separately. In each run, while performing the pRF experiments, participants fixated monocularly on a small circular disk that appeared in the center of their visual field and changed in color between red and green. To ensure their central fixation and attention to the experiment, subjects were asked to respond every time the small circular disk in the center changed color by using an MRI-compatible response device.

### MRI Data Preprocessing

All pRF data were analyzed using the Analysis of Functional NeuroImages (AFNI) software package^32^ (provided in the public domain by the National Institutes of Health, Bethesda, MA, USA; http://afni.nimh.nih.gov/afni). Images were preprocessed to control for subject motion (3dvolreg) by using the first volume of the first run as a reference, after removing the first four volumes to establish equilibrium. After motion-correction, images were detrended (3dDetrend, removing second-order polynomial trends) and smoothed (3dmerge) with a 5-mm full-width at half-maximum Gaussian kernel. Given that a single ∼3-minute run was acquired per eye, time series data were smoothed to boost signal-to-noise. Notably, unsmoothed time series were also analyzed and did not change the overall pattern of results and the correlations between clinical and pRF measurements.

### pRF Analysis

All pRF analyses were conducted in AFNI by using a pRF implementation for the AFNI distribution, developed by Richard Reynolds.^33^ For every voxel in the brain, the model initially estimates the center of the pRF on an x, y grid with 200 samples across both the height and width of the field of view. For each point in the grid, the sizes of pRFs (sigma) are sampled at the same resolution but over a default range of 0 to half the field of view (sampled at 100 even intervals). These default parameters result in four million possible pRFs (with unique x, y location and size). Given the position of the stimulus in the visual field at every TR, the estimated time series for a receptive field of a given location and size is modeled. The model then makes use of a 2-D stimulus time series, which contains binary masks of the stimulus location at each TR and a convolution with a standard hemodynamic response function to produce four million predicted time series. Both Simplex and Powell optimization algorithms are used simultaneously to find the best time series/parameter sets (x, y, and size [sigma]) by minimizing the least-squares error of the predicted time series measured against the acquired time series in each voxel. The model outputs for each voxel include the estimated diameter for the pRFs (size/sigma), along with the x, y locations representing the center of the pRF, and the corresponding explained variance (R^2^) for the fit, which can be used to statistically threshold these data.

### Visual Field Coverage

The visual field coverage maps were computed individually for all participants (controls and patients). These maps were derived from all voxels, with an explained variance >20%. Each voxel's pRF was plotted as a scaled 2-D Gaussian onto a matrix representing the visual field. Once all pRFs were overlaid, the maximum explained variance (across voxels) was calculated for every position in the visual field (pixel of the matrix).^14,33^ To normalize visual field coverage plots across all participants, these values were divided by the maximum explained variance that was present in one of the HC participants (HC1).

### Surface Reconstructions

Surface reconstructions of the gray and white matter boundary of individual hemispheres for each participant were made using the Freesurfer4 autorecon script (provided in the public domain, http://surfer.nmr.mgh.harvard.edu/) and visualized using the Surface Mapping with AFNI (SUMA) software package.

## Results

### Clinical Characterization of CHM

**Figure 1:**
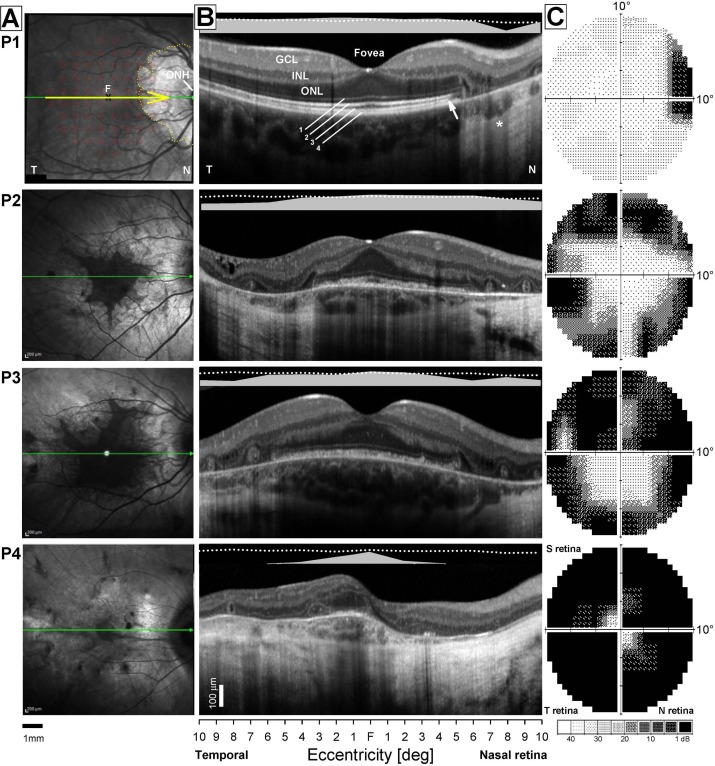
Central retinal structural and functional changes in the patients. (A) NIR-REF images of the central retina. Images are 30° × 30° centered at the fovea (F). Horizontal green arrows show the position and direction of the SD-OCT scans; overlaid yellow arrow in P1 shows a 6-mm segment used for correlation between SD-OCT and function in B. Dotted yellow line demarcates a pale area of chorioretinal atrophy near the optic nerve head (ONH) that is surrounded by retina with a grossly normal NIR-REF appearance. Overlaid grid of red circles shows the position and size of the visual stimuli (n = 68) used to test the sensitivity of the central retina with the 10-2 protocol in C. Scale bar at bottom. (B) Shown are 6-mm-long horizontal SD-OCT cross sections from temporal (T) through the fovea into nasal (N) retina in the four CHM patients. Scans cover region shown with horizontal yellow arrow in A. Nuclear layers (outer nuclear layer, ONL; inner nuclear layer, INL; ganglion cell layer GCL) are labeled in the temporal retina of P1, which shows a normal retinal architecture. Outer photoreceptor/RPE laminae are numbered (1, outer limiting membrane; 2, inner segment/outer segment region or ellipsoid zone (EZ); 3, interdigitation zone (IZ) between photoreceptor outer segment tips and the apical RPE; 4, RPE/Bruch's membrane [RPE/BrM]). Arrow points to a region closest to the fovea where there is loss of the IZ signal (or photoreceptor outer segment tip), which signals the beginning of a transitional zone (TZ) of increasing structural abnormalities with increasing eccentricity from the foveal center. Apteryx points to region of increased backscattering of the SD-OCT signal caused deeper penetration of the signal through depigmented RPE outside of the TZ. Bars above the scans show psychophysically determined light-adapted sensitivities along a horizontal profile that colocalizes with the region scanned with SD-OCT. Dotted lines above bars define the lower limit (normal mean, 2 SD) of the sensitivity estimates. T, temporal retina; N, nasal retina. Calibration bar to the bottom left. (C) Central visual field sensitivities measured with automated light-adapted static threshold perimetry and a conventional system and clinical protocol (Humphrey Field Analyzer; 10-2 protocol). Maps extend to 10° of eccentricity and are flipped vertically to match the corresponding retinal position of the test grid on the NIR-REF images. Raw sensitivity estimates are plotted as gray scale maps (bottom of column); black areas represent absolute scotomas to this testing protocol (sensitivities ≤0 dB), lighter areas represent better (>30 dB) sensitivities.

The CHM patients enrolled in this study (Table 1) exemplify different extents of relative histologic preservation at a stage of the disease when this otherwise predominantly midperipheral disease reaches the central retina (Fig. 1). On infrared reflectance (NIR-REF) imaging, the patient with the mildest abnormalities (patient 1, P1) shows a lighter region near the optic nerve, with a scalloped contour (Fig. 1A, yellow dotted line). The rest of the central retina has a normal NIR-REF appearance. An SD-OCT cross section through the foveal center shows a normal lamination pattern in most of the scan (Fig. 1B). Nasal to the fovea, coinciding with the boundary between normal and hyperreflective NIR-REF signal, there is a transition zone (TZ) of abrupt changes in thickness and organization of the retinal layers. There is an orderly loss of structures with increasing distance from the fovea: first, there is loss of the signal that originates from the interdigitation zone between the photoreceptor outer segment tips and the apical retinal pigment epithelium (RPE), followed by attenuation and loss of the zone of transition between the photoreceptor inner segment and the photoreceptor outer segment or ellipsoid zone, and ending with thinning and loss of the outer nuclear layer. Nasal to the TZ (Fig. 1B, P1, arrow), increased deep backscattering of the SD-OCT signal caused by RPE depigmentation and/or loss (Fig. 1B, P1, asterisk) precedes a region of total chorioretinal atrophy. Colocalized light-adapted sensitivity profiles are normal across most of the length of the scan, except for localized loss within and nasal to the TZ (Fig. 1B). The rectangular grid of the standard 10-2 SAP protocol was used to determine the topographic distribution of the sensitivity losses across the central 10° field in the patients (Fig. 1A, C). A sensitivity map shows a dense (≥50-dB loss) scotoma at ∼6° in nasal retina that corresponds to the TZ of structural change. Severe sensitivity loss at this location is surrounded by retina with normal or near-normal light-adapted sensitivity, a common feature in CHM. With disease progression, the centripetal movement of the TZs leaves residual geographic islands of relative preservation of the NIR-REF signal and underlying structure on SD-OCT, surrounded by chorioretinal atrophy (Figs. 1, P2–P4). Even at these stages, cone-mediated vision may be near normal at the foveal center but transitions abruptly to a blind atrophic retina. The associated topography of the remaining central visual field matches the shape of the residual islands on NIR-FAF (Figs. 1A,C). In every example, the disease “dissects” out regions of relative structural and functional preservation from the surrounded blind retina. The pattern presents a unique platform in which to explore the relationships between the topography of the retinal structure and function and its cortical representation by fMRI.

### Visual Field Coverage From pRF Modeling

To compare clinical and pRF measurements for each CHM and HC participant, we initially computed visual field coverage maps from all significantly modulated voxels (>20% explained variance), pooled across both hemispheres. The inclusion of voxels from both hemispheres matches the SAP measurements, as both hemispheres process visual signals from each eye.

**Figure 2:**
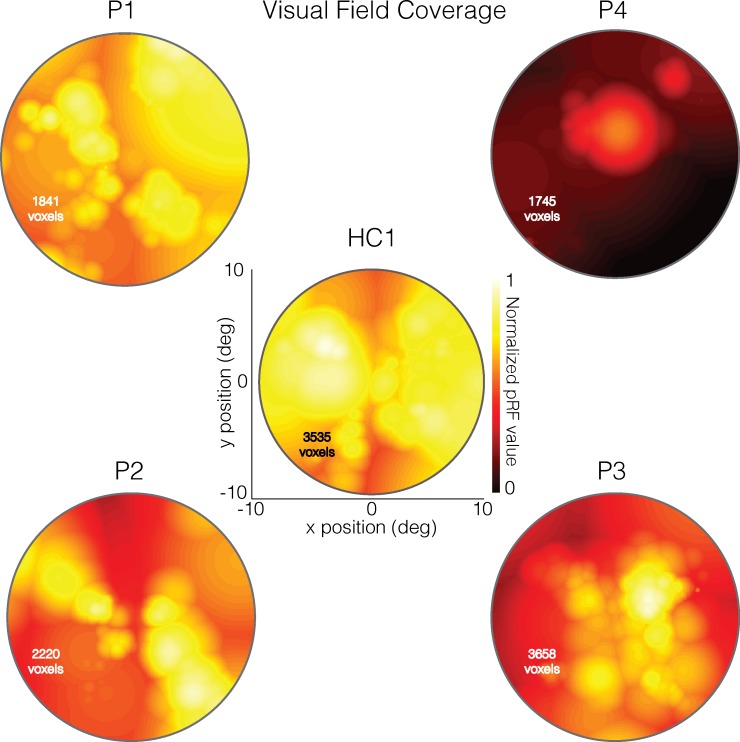
Left eye pRF visual field coverage maps for four CHM participants and one HC (HC1). To compare the results with individual clinical assessments, visual field data were restricted to +10° and −10° of the entire visual field. These plots represent the maximum pRF coverage at each position in the visual field and were derived from all significantly modulated voxels (>20% explained variance), pooled across both hemispheres. The total number of included voxels is inset onto each map. As shown in the center image, the visual field coverage for a typical HC (HC1) exhibits high pRF values (hot colors) across the visual field except at the extreme upper and lower vertical meridians. Coverage plots for CHM participants are presented relative to the maximum pRF intensity in HC1. As shown here, P1, who had relatively preserved peripheral and central vision, presented with a similar pRF distribution to HC1, albeit patchier and with slightly reduced intensity. Unlike P1, P4 was at a more advanced stage of disease progression, with much more restricted peripheral vision and much smaller preserved central vision. The visual field coverage for P4 is strikingly different from HC1 as well as P1. The two remaining CHM participants (P2 and P3) represent mid-stage disease, and the pRF visual field coverage for these CHM participants are also strikingly different from that of HC1. However, both of these CHM participants show greater degrees of central and peripheral vision as compared with the pRF distribution for P4. P2 showed relatively high pRF values in the upper left and lower right quadrants of the visual field, whereas P3 showed a strong central representation extending further into the lower visual field and a markedly reduced representation of the periphery.

Visual field coverage maps represent the maximum pRF value at each location in the visual field and are displayed for the left eye data of each CHM participant and a representative HC participant (HC1) in Figure 2 (See Supplementary Fig. S2 for the right eye data). These visual field coverage maps clearly depict a more restricted peripheral visual field, with the presence of a more centralized vision in CHM participants (Fig. 2). The strength of peripheral representations reflects the various disease stages in this sample of CHM participants, with the weakest peripheral representations observed for participants in mid- to late-stage disease (e.g., P3, P4). In particular, P4, with end-stage disease, exhibits only central visual field representations and little or no contribution from more peripheral locations.

### pRF Visual Field Coverage Maps Correlate with SAP Measurements in CHM

**Figure 3:**
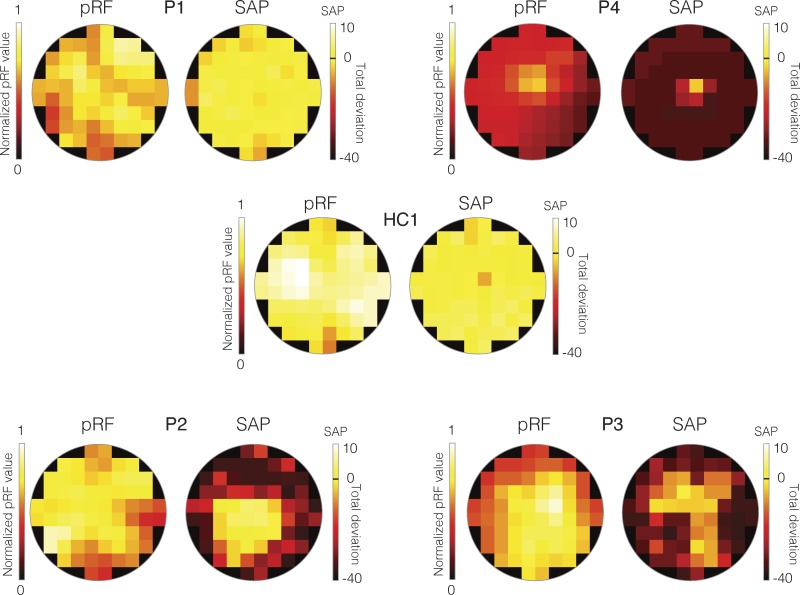
Total deviation SAP and modified pRF visual field coverage maps for the left eye data from all patients and a representative HC (HC1). In each case, the modified pRF visual field coverage maps are displayed as heat maps to the left. These visual field coverage maps have been normalized to the maximum value within the modified visual field coverage map of HC1. Positions in the visual field with higher pRF values are color-coded as yellow/white, with positions in the visual field with lower pRF values color-coded as red/black. The right-hand plot in each case depicts the total deviation SAP measurements as heat maps. In these plots, bright cells (yellow/white) represent locations in the visual field with sensitivity either equal to or greater than normal age-matched controls, whereas dark-colors (red/maroon) represent locations in the visual field with decreased sensitivity relative to a normal age-matched control. The black-line on the color bar represents the same sensitivity as a normal age-matched control. Thus, bright yellow/white cells indicate increased sensitivity and orange/red/maroon cells indicate decreased sensitivity.

To quantify the relationship between SAP and pRF measurements, visual field coverage maps were spatially resampled to match the SAP measurements (see Supplementary Material). The SAP and resulting modified pRF visual field coverage maps for the left eye of each CHM participant and a representative control (HC1) are displayed in Figure 3. Visual inspection of Figure 3 demonstrates clear similarities between the SAP and pRF measurements for CHM participants, with similar heat map intensity patterns for cells occupying largely commensurate locations. For instance, in P4, the highest SAP and pRF values are located at the very center of the visual field in both measurements (Fig. 3, top right). Similarly, the lower left quadrant of the visual field and the right visual field contain both the highest SAP and pRF values in P2 and P3, respectively (Fig. 3, bottom).

**Figure 4:**
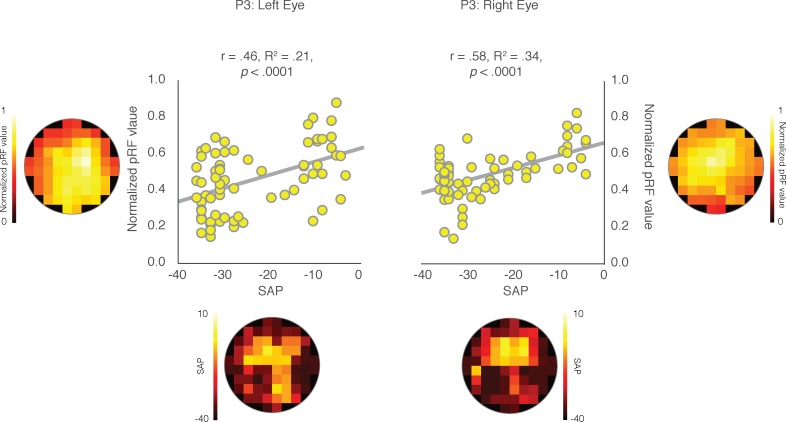
Pearson's correlation between clinical and pRF measurements for both eyes in P3. As shown on the left, the mean pRF values for each cell location is presented on the y-axis and the corresponding total deviation value, taken from the SAP, is shown on the x-axis. For ease of visual comparison, the calculated pRF and total deviation heat-maps are shown along the “Y” and “X” axes, respectively. The Pearson's correlations between these two measures are plotted for all 68 cells for the left and right eye separately. Significant positive correlations were observed between the SAP and pRF measures for both the left (r = 0.46; P < 0.0001) and the right (r = 0.58; P < 0.0001) eye, demonstrating strong associations between the two measures of visual function in P3.

The Pearson's correlation between the two measurements was computed for each CHM and HC participant and is displayed for both the left and right eyes of P3 in Figure 4, along with the SAP and modified pRF visual field coverage maps. As shown in Figure 4, significant and positive correlations between SAP and pRF measurements were observed for both the left (r = 0.46, *P* < 0.0001) and right (r = 0.58, *P* < 0.0001) eyes in P3. Indeed, positive and significant relationships between SAP and pRF measurements were present in all but two cases (P1, right eye and P2; right eye) among CHM participants. In contrast, HCs showed limited consistency in the relationship between SAP and pRF measurements (Table 2).

**Table 2:**
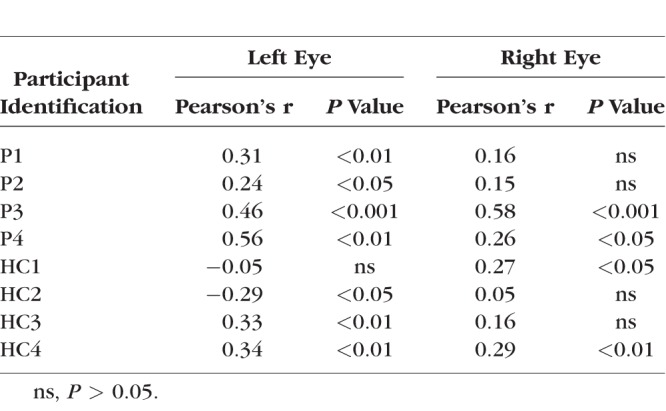
Pearson's Correlation Values Between SAP and pRF Measurements and Corresponding P values for the Left and Right Eyes of Both Patients and HCs

### pRF Strength Across Eccentricity Reflects Clinical Eccentricity Profiles

**Figure 5:**
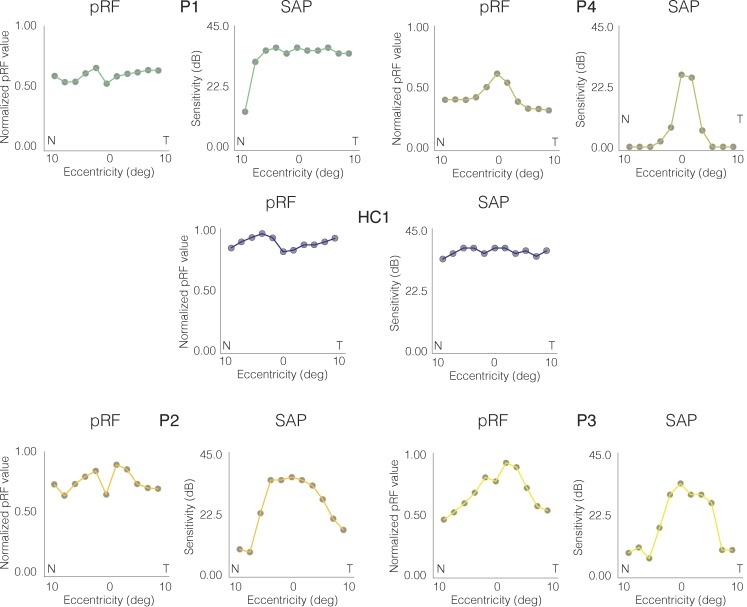
Clinical and pRF horizontal eccentricity profiles for the left eye of all CHM participants and a representative HC (HC1). In each case, the pRF derived horizontal eccentricity is plotted to the left, with the clinically derived visual sensitivity plotted to the right. Across all plots, horizontal position (deg) is plotted on the x-axis, with the nasal (N) and temporal (T) visual fields labeled. For pRF profiles, the pRF value at that position is plotted on the y-axis, normalized to the maximum pRF value within HC1. For clinical profiles, visual sensitivity (dB) is plotted on the y-axis.

To quantify the relationship between clinical and pRF eccentricity profiles, visual field coverage maps were spatially resampled to match the spatial sampling of clinical assessments (see Supplementary Material). The clinical and resampled pRF eccentricity profiles for the left eye of each CHM participant and a representative control (HC1) are displayed in Figure 5. As shown in Figure 5, clinical and pRF eccentricity profiles show strikingly similar patterns in mid (P2, P3) and late (P4) stage CHM participants, with lower sensitivity (poorer vision/lower pRF value) at eccentric positions in both nasal and temporal fields and a sharp rise in sensitivity (better vision/higher pRF value) toward the center of the visual field (eccentricity, 0°). The similarity in eccentricity profiles is particularly clear for P4 with end-stage disease. In both measurements, sensitivity values are largely flat across eccentricity, apart from a sharp rise at the center of the visual field (Fig. 5, top right).

**Figure 6:**
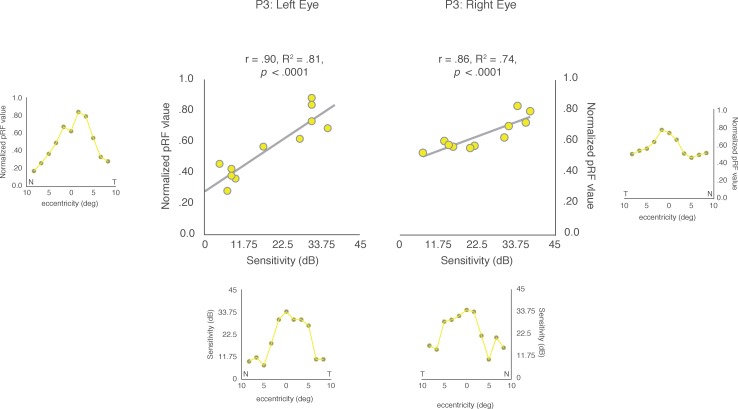
Pearson's correlation between clinical and pRF eccentricity profiles for both eyes in P3. For ease of visual comparison, the pRF eccentricity profiles are shown next to the y-axes and the values for the clinical eccentricity profiles (recreated from the clinical data) are presented below the x-axes, for both the left and right eyes, respectively. The Pearson's correlations between these two measures are plotted for both the left and right eyes. Significant and positive correlations were observed for both the left (r = 0.90; P < 0.0001) and right (r = 0.86; P < 0.0001) eyes, respectively.

The Pearson's correlation between the two measurements of horizontal eccentricity was computed separately for the left and right eyes for each CHM and HC participant. An example of the correlation results for P3 is shown in Figure 6. We observed highly significant positive correlations between clinical and pRF eccentricity profiles for the left (r = 0.90, *P* < 0.0001) and right (r = 0.86, *P* < 0.0001) eyes in P3. The strong positive relationship between clinical and pRF eccentricity profiles was also present for both the left and right eyes of P2 and P4, but not P1 who was at an early disease stage (see Table 3 for statistical breakdown). In contrast to the CHM participants, HCs presented with clinical eccentricity profiles that were, for the most part, flat across the entire extent of the tested central visual field, which likely contributes to the largely nonsignificant and inconsistent relationships between clinical and pRF eccentricity profiles across HCs (Supplementary Fig. S4).

**Table 3:**
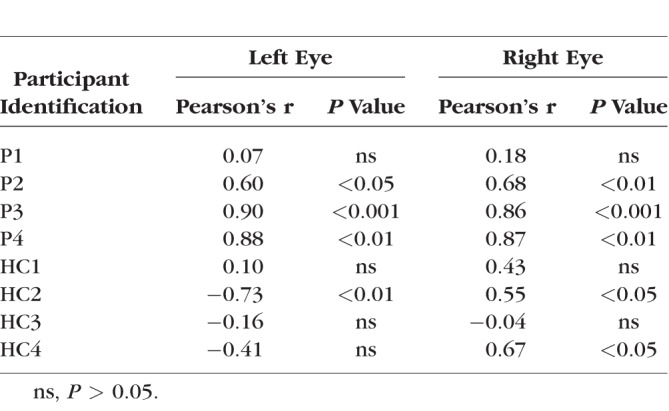
Pearson's Correlation Values Between Clinical and pRF Eccentricity Profiles and Corresponding P values for the Left and Right Eyes of Patients and HCs

## Discussion

The peculiar topography of the visual field and the depth of the sensitivity loss of patients with CHM in this study were well represented in the spatial distribution and strength of pRF estimates. Importantly, the strength of this positive relationship increased with increasing stages of disease progression. Indeed, this positive relationship was most prominent in CHM participants at moderately advanced or late disease stages and was either less consistent or not present in HCs or participants at the earliest stages of disease progression, respectively.

Links between visual function, as measured clinically with SAP, and cortical representations, as measured through fMRI^34^ and specifically pRFs,^14^ have been reported for patients with cortical lesions.^14^ and in patients with Leber's congenital amaurosis following retinal gene therapy.^31^ This study used CHM as a disease model of photoreceptor degeneration and demonstrated strong positive relationships between SAP and pRF measurements. The current data are consistent with many previous fMRI studies of diverse patient populations^10,14,34–35^ that have similarly demonstrated a link between clinical measures of visual function and cortical responses measured with fMRI.

Previous work has used fMRI^36–38^ and, in particular, pRF modeling^11^ to investigate potential mechanisms of foveal reorganization in patients with macular degeneration, with reports both for^36–38^ and against^11^ the technique. Here, the focus was on demonstrating the relationship between the clinical measures of visual function and cortical representations of the visual field in patients with CHM and not on potential mechanisms of cortical reorganization^39^ caused by disease. The potential for cortical reorganization in CHM patients is, however, an important topic that should be the focus of future studies, especially given the existing evidence of retinal remodeling in CHM as well as the somewhat unusual longevity of high levels of fine spatial discrimination in this condition.^22,29^

Significant and positive correlations were observed between clinical and pRF eccentricity profiles in all but one CHM participant (P1 left and right eyes) who was at an early disease stage. Thus, as compared to other CHM participants who were at later stages of disease progression, P1 presented with near-normal horizontal eccentricity profiles (See Supplementary Fig. S4). As such, similarly to HC participants, the correlations between SAP and pRF measurements in P1 could have been highly affected by the lack of variation in visual field sensitivity. In all other CHM cases, strong positive correlations were observed, demonstrating that the strength of pRFs as a function of horizontal eccentricity strongly predicts the sensitivity of vision at those same eccentricities measured clinically. The lack of consistent positive correlations between clinical and pRF measurements in P1 and in all HCs may reflect a ceiling effect in both measurements. For instance, the range of SAP values across HCs (15; max, 4; min, −11) was less than half of the range across participants with CHM (41; max, 5; min, −36). The second analysis, which focused on visual sensitivity as a function of horizontal eccentricity, is also likely affected by a similar ceiling effect. The sensitivity profiles of the HCs, as measured herein with a relatively large (0.45° diameter) achromatic stimulus, were mainly flat across the entire width of the tested visual field, with a maximum range of only 6 dBs (Supplementary Fig. S4). Such constant sensitivity across the visual field likely impacted the ability to observe consistent and significant correlations in the HCs. The visual field stimuli used in this work was intended to match protocols commonly used in the clinic. However, in mildly affected early-stage patients and HCs, this model may not adequately represent the variation within the visual field to produce a more sensitive relationship with the pRF measures. The steep transitions in retinal tissue and visual function expressed in CHM patients at mid- and late-disease stages, likely contribute to the ability of the pRF method to better identify such variability. As such, the pRF methodology may be less suited for detection of early-stage disease in patients with slow retinal degeneration.

Data from pRF estimates, which can be acquired in a single ∼3-minute fMRI session, may provide additional objective information for the functional evaluation of patients with inherited retinal degenerations, particularly for those that cause sharp transitions in visual function, such as CHM. Notably, previous studies^14,40^ have suggested that pRF estimates can be biased when a predictable stimulus is used such as in this study. However, to the extent that any bias is present in our data would only serve to reduce the correlation between SAP and pRF measurements and cannot explain the positive correlations that were observed. Moreover, these data provide compelling evidence for the feasibility of using pRF estimates as efficient and objective outcome measures for investigating potential cortical effects of interventions to restore vision, such as retinal gene therapy. The current data demonstrate the supportive role that pRF estimates can play in complimenting patients' ophthalmic examinations. The advantages of using pRFs as complementary outcome measures are that they are efficient and objective estimates of visual sensitivity that accurately reflect brain function. All existing pRF models output, for each voxel in the brain, a series of parameter estimates whose values are interpretable with respect to the tested visual field. The importance of fMRI and, in particular, pRF measurements may be amplified when used in conjunction with ongoing clinical assessments of interventions to restore vision, such as retinal gene therapy.^34,41–42^ Indeed, studies assessing the effect of retinal gene therapy on visual function in patients with CHM^41–42^ report improved rod and cone functions accompanied with a gain in visual acuity of the treated eye that correlated with the vector dose administered. This improvement in visual function was also reported in two patients at 3.5-years posttreatment.^42^ It remains to be seen whether pRF measurements are sensitive enough to detect any visual function improvements following retinal gene therapy interventions.

## Conclusions

Although reflecting only a small sample size, results from this report demonstrate that pRF estimates in the visual cortex correlate positively and significantly with clinical measures of visual function in patients with CHM. In particular, pRF measurements accurately mapped the topography of the field of vision in CHM. Most importantly, the strength of the correlation between clinical and pRF measurements increased with disease severity. Unlike most CHM participants, HCs presented with inconsistent correlations between clinical assessments and pRF measures, which likely reflect ceiling effects in both measurements. This preliminary study shows the potential use of pRF estimates as an objective tool complementary to clinical measures of vision and to explore the consequences that retinal degenerations and their treatments have on visual processing beyond the retina and into the brain.

## Supplementary Material

Supplement 1

## References

[1] Wandell BA, Dumoulin SO, Brewer AA (2007). Visual field maps in human cortex. *Neuron*.

[2] DeYoe EA, Bandettini P, Neitz J, Miller D, Winan P (1994). Functional magnetic resonance imaging (FMRI) of the human brain. *J Neurosci Methods*.

[3] Sereno M, Dale AM, Reppas JB (1995). Borders of multiple visual areas in humans revealed by functional magnetic resonance imaging. *Science*.

[4] Engel SA, Glover GH, Wandell BA (1997). Retinotopic organization in human visual cortex and the spatial precision of functional MRI. *Cereb Cortex*.

[5] Dumoulin SO, Wandell BA (2008). Population receptive field estimates in human visual cortex. *Neuroimage*.

[6] Lee S, Papanikolaou A, Logothetis NK, Smirnakis SM, Keliris GA (2013). A new method for estimating population receptive field topography in visual cortex. *Neuroimage*.

[7] Kay KN, Winawer J, Mezer A, Wandell BA (2013). Compressive spatial summation in human visual cortex. *J Neurophysiol*.

[8] Wandell BA, Winawer J (2015). Computational neuroimaging and population receptive fields. *Trends Cogn Sci*.

[9] Sunness JS, Liu T, Yantis S (2014). Retinotopic mapping of the visual cortex using functional magnetic resonance imaging in a patient with central scotomas from atrophic macular degeneration. *Ophthalmology*.

[10] Schumacher EH, Jacko JA, Primo SA (2008). Reorganization of visual processing is related to eccentric viewing in patients with macular degeneration. *Restor Neurol Neurosci*.

[11] Baseler HA, Gouws A, Haak KV (2011). Large-scale remapping of visual cortex is absent in adult humans with macular degeneration. *Nat Neurosci*.

[12] Duncan RO, Sample PA, Weinreb RN, Bowd C, Zangwill LM (2007). Retinotopic organization of primary visual cortex in glaucoma: a method for comparing cortical function with damage to the optic disk. *Invest Ophthalmol Vis Sci*.

[13] Masuda Y, Horiguchi H, Dumoulin SO (2010). Task-dependent V1 responses in human retinitis pigmentosa. *Invest Ophthalmol Vis Sci*.

[14] Papanikolaou A, Keliris GA, Papageorgiou TD (2014). Population receptive field analysis of the primary visual cortex complements perimetry in patients with homonymous visual field defects. *Proc Natl Acad Sci U S A*.

[15] Cremers FP, van de Pol DJ, van Kerkhoff LP, Wieringa B, Ropers HH (1990). Cloning of a gene that is rearranged in patients with choroideraemia. *Nature*.

[16] Merry DE, Jänne PA, Landers JE, Lewis RA, Nussbaum RL (1992). Isolation of a candidate gene for choroideremia. *Proc Natl Acad Sci U S A*.

[17] van Bokhoven H, van den Hurk JA, Bogerd L (1994). Cloning and characterization of the human choroideremia gene. *Hum Mol Genet*.

[18] van den Hurk JA, Schwartz M, van Bokhoven H (1997). Molecular basis of choroideremia (CHM): mutations involving the Rab escort protein-1 (REP-1) gene. *Hum Mutat*.

[19] Seabra MC, Brown MS, Slaughter CA, Südhof TC, Goldstein JL (1992). Purification of component A of Rab geranylgeranyl transferase: possible identity with the choroideremia gene product. *Cell*.

[20] Seabra MC, Ho YK, Anant JS (1995). Deficient geranylgeranylation of Ram/Rab27 in choroideremia. *J Biol Chem*.

[21] Seabra MC, Mules EH, Hume AN, GTPases Rab (2002). intracellular traffic and disease. *Trends Mol Med*.

[22] Aleman TS, Han G, Serrano LW (2017). Natural history of the central structural abnormalities in choroideremia: a prospective cross-sectional study. *Ophthalmology*.

[23] McCulloch C, McCulloch RJ (1948). A hereditary and clinical study of choroideremia. *Trans Am Acad Ophthalmol Otolaryngol*.

[24] Kärnä J (1986). Choroideremia: a clinical and genetic study of 84 Finnish patients and 126 female carriers. *Acta Ophthalmol Suppl*.

[25] Hayakawa M, Fujiki K, Hotta Y (1999). Visual impairment and REP-1 gene mutations in Japanese choroideremia patients. *Ophthalmic Genet*.

[26] Duncan JL, Aleman TS, Gardner LM (2002). Macular pigment and lutein supplementation in choroideremia. *Exp Eye Res*.

[27] Roberts MF, Fishman GA, Roberts DK (2002). Retrospective, longitudinal, and cross sectional study of visual acuity impairment in choroideraemia. *Br J Ophthalmol*.

[28] Jacobson SG, Cideciyan AV, Sumaroka A (2006). Remodeling of the human retina in choroideremia: rab escort protein 1 (REP-1) mutations. *Invest Ophthalmol Vis Sci*.

[29] Nabholz N, Lorenzini MC, Bocquet B (2016). Clinical evaluation and cone alterations in choroideremia. *Ophthalmology*.

[30] Jacobson SG, Voigt WJ, Parel JM (1986). Automated light- and dark-adapted perimetry for evaluating retinitis pigmentosa. *Ophthalmology*.

[31] Ashtari M, Cyckowski L, Yazdi A (2014). fMRI of retina-originated phosphenes experienced by patients with Leber congenital amaurosis. *PLoS One*.

[32] Cox RW (1996). AFNI: software for analysis and visualization of functional magnetic resonance neuroimages. *Comput Biomed Res*.

[33] Silson EH, Chan AWY, Reynolds RC, Kravitz DJ, Baker CI (2015). A retinotopic basis for the division of high-level scene processing between lateral and ventral human occipitotemporal cortex. *J Neurosci*.

[34] Ashtari M, Nikonova ES, Marshall KA (2017). The role of the human visual cortex in assessment of the long-term durability of retinal gene therapy in follow-on RPE65 clinical trial patients. *Ophthalmology*.

[35] Morland AB, Baseler HA, Hoffmann MB, Sharpe LT, Wandell BA (2001). Abnormal retinotopic representations in human visual cortex revealed by fMRI. *Acta Psychol*.

[36] Baker CI, Dilks DD, Peli E, Kanwisher N (2008). Reorganization of visual processing in macular degeneration: replication and clues about the role of foveal loss. *Vis Res*.

[37] Dilks DD, Julian JB, Peli E, Kanwisher N (2014). Reorganization of visual processing in macular degeneration depends on foveal loss. *Optom Vis Sci*.

[38] Baseler HA, Brewer AA, Sharpe LT, Morland AB, Jägle H, Wandell BA (2002). Reorganization of human cortical maps caused by inherited photoreceptor abnormalities. *Nat Neurosci*.

[39] Wandell BA, Smirnakis SM (2009). Plasticity and stability of visual field maps in adult primary visual cortex. *Nat Rev Neurosci*.

[40] Binda P, Thomas JM, Boynton GM, Fine I (2013). Minimizing biases in estimating the reorganization of human visual areas with BOLD retinotopic mapping. *J Vis*.

[41] MacLaren RE, Groppe M, Barnard AR (2014). Retinal gene therapy in patients with choroideremia: initial findings from a phase 1/2 clinical trial. *Lancet*.

[42] Edwards TL, Jolly JK, Groppe M (2016). Visual acuity after retinal gene therapy for choroideremia. *N Engl J Med*.

[43] Masuda Y, Dumoulin SO, Nakadomari S, Wandell BA (2008). V1 projection zone signals in human macular degeneration depend on task, not stimulus. *Cereb Cortex*.

