# Supplementary material for: Comparing Clinical Perimetry and Population Receptive Field Measures in Patients with Choroideremia
Source: Invest Ophthalmol Vis Sci. 2018 Jul;59(8):3249–58. doi: 10.1167/iovs.18-23929 (PMC6110169; doi:10.1167/iovs.18-23929)
Supplement: Supplement 1 [file iovs-59-07-23_s01.pdf]

Static Automatic Perimetry Maps

Comparisons between clinical and pRF measurements were made initially with respect to standard static automatic perimetry (SAP) total deviation maps. These maps, depicted as heat-maps for all CHM and HC participants in Supplementary Figure 1, demonstrate the visual sensitivity of an individual as compared to the visual sensitivity of a normal age-matched control. In these plots, bright-colors (yellow/white) represent locations in the visual field that are either as sensitive or better than a normal age-matched control, whereas dark-colors (red/maroon) represent location in the visual field with decreased sensitivity from a normal age-matched control. As expected, all HCs demonstrate normal or near normal visual sensitivity across the tested visual field. In each HC, the majority of the SAP maps are bright, which demonstrates similar sensitivity to a normal age-matched control. In contrast, the dramatic reduction in peripheral sensitivity is clearly evident for CHM participants (P2-P4), with dark-colored cells (poor visual sensitivity) in the periphery. For example, P4 who was at a late disease stage exhibits only moderately bright cells at the very center of the visual field for both the left and right eyes. P1, who represents early stages of disease progression exhibits relatively normal visual field sensitivity, particularly for the left eye. In the right eye, the fair right visual field has poorer sensitivity.

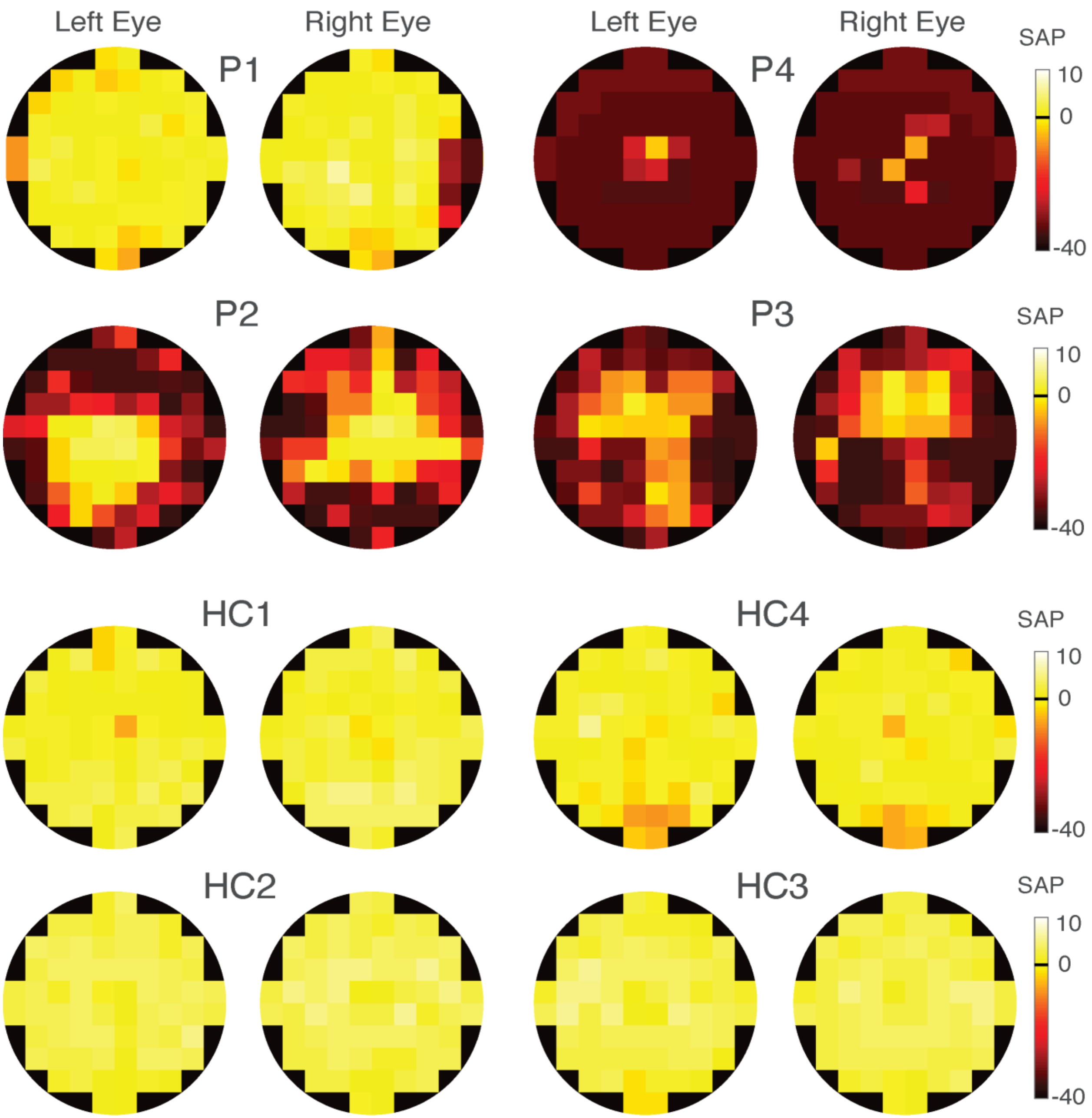

Supplementary Figure 1. Total deviation SAP maps for each CHM participant, HC and eye, respectively. Total deviation maps represent the level of visual sensitivity relative to a normal age-matched control. In these plots, bright-cells (yellow/white) represent locations in the visual field with sensitivity either equal or greater than normal age-matched controls, whereas dark-colors (red/maroon) represent locations in the visual field with decreased sensitivity relative to a normal age-matched control. The black-line on the color-bar represents the same sensitivity as

a normal age-matched control. Thus, bright yellow/white cells indicate increased sensitivity and orange/red/maroon cells indicate decreased sensitivity.

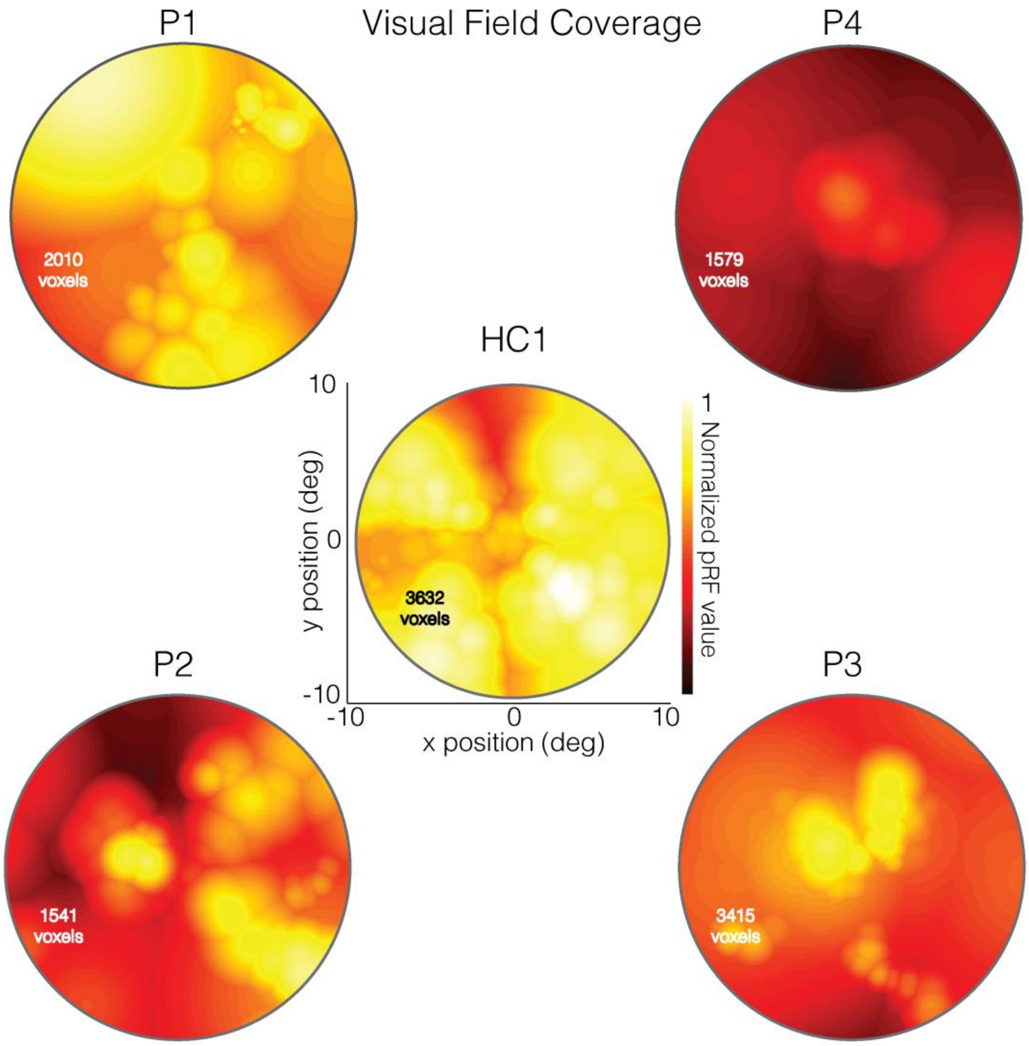

Supplementary Figure 2. Right eye pRF visual field coverage plots for four CHM participants and one healthy control (HC1). Similar to the left eye, assessment of the visual fields was restricted to + 10 and -10 degrees of the entire visual field. The pRF distribution for a typical healthy control (HC1) is presented in the center image. As expected, similar to the left eye, HC1 presented with high pRF values across the visual field with relatively high intensity levels except at the extreme upper and lower vertical meridians, a pattern regularly observed in pRF mapping. Coverage plots for CHM participants' right eye were assessed relative to HC1's maximum pRF intensity levels. pRF distribution for the CHM participant with most vision (P1) showed similarity

to HC1 with higher intensities in the upper left visual field but great reduction of visual intensities in the lower left quadrant. The CHM participant with more advanced disease and limited visual field (P4) showed small centrally located pRF distribution with moderate intensity values. The two CHM participants in mid disease stages (P2 and P3) both showed dramatically more attenuated visual intensities for their right eye pRF distributions as compared to HC1. At the same token, their pRF distributions also differed dramatically from each other. While P2 presented with increase central and right peripheral visual field, P3's visual field coverage was mainly limited to a strong central representation, which was largely restricted to the upper visual field.

Quantification of Relationship Between SAP and pRF Measurements

To quantify the relationship between SAP measurements and pRF parameters, visual field coverage maps needed to be resampled to match the spatial sampling of SAP measurements. Thus, based on the SAP of a 10-2 (10x10 matrix), visual field coverage maps were divided evenly into 10x10 square grids, with each grid representing 2x2 degrees of visual angle (Supplementary Figure 3A, 3B). Next, the average pRF values within each grid were calculated, normalized to the maximum of a representative control (HC1) and color-coded as heat maps (Supplementary Figure 3C, 3D for P3). This process was repeated for all participants and their left and right eyes, separately**.** Since from the 100 cells of the 10x10 SAP matrix only 68 were supplemented with visual data**,** visual field coverage maps were also evaluated for the same exact 68 cell locations matching those sampled during SAP measurements. Once each cell was populated with both the SAP and pRF values the Pearson's correlation (r) between the two measurements was derived for all participants and for each eye separately.

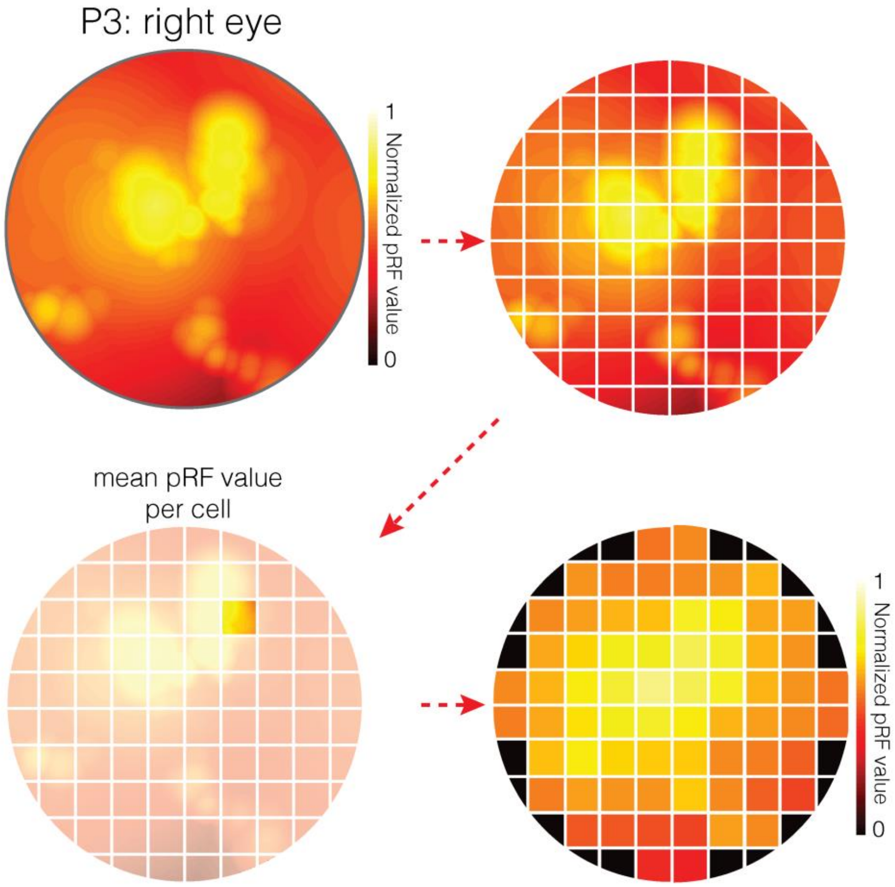

Supplementary Figure 3. Schematic for quantifying the relationship between SAP and pRF measurements. **(A)** The visual field coverage map derived for the right eye of P3 is shown as an example. **(B)** Following the Total Deviation analysis from the SAP measurement (10x10 grid: 68 data cells), the visual field coverage map was divided evenly into a 10x10 grid. **(C)** Each individual cell was assigned the mean pRF value for that location. The same 68 visual field locations as sampled in SAP measurements were selected. **(D)** The mean pRF value in each cell was normalized to the maximum grid cell in a representative control (HC1).

Horizontal Eccentricity Profiles

The second comparison between clinical and pRF measurements were made with respect to visual sensitivity as a function of horizontal eccentricity. The clinical eccentricity profiles for all CHM and HC participants and their left and right eyes are plotted in Supplementary Figure 4. Unlike the majority of participants with CHM, who exhibit clear decreases in sensitivity in the periphery of both nasal and temporal fields (P2-P4), eccentricity profiles for HCs are largely flat across the entire extent of the tested visual field. This lack of variability likely impacted the outcome of inconsistent correlations with the pRF measurements in the HCs.

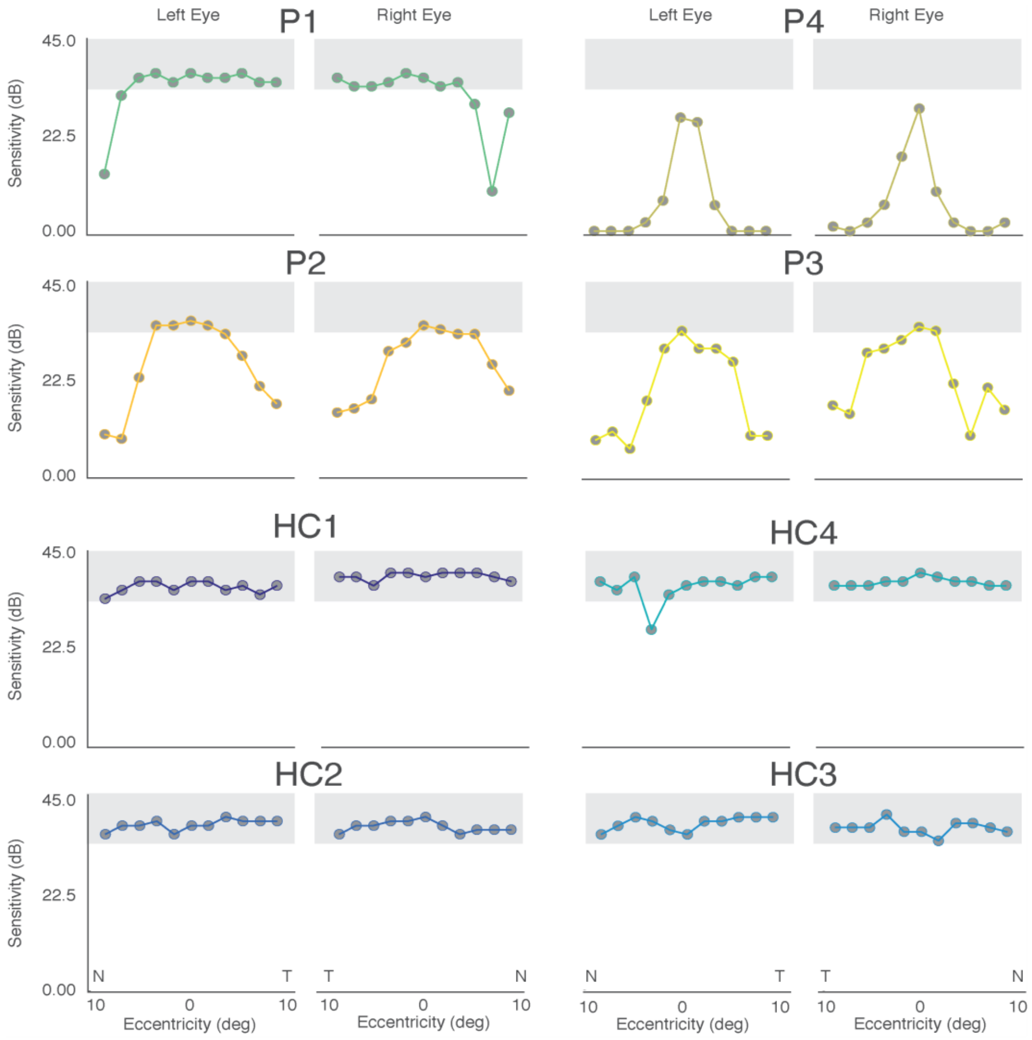

Supplementary Figure 4. Clinical eccentricity profiles for CHM and HC participants. The clinical measurements of horizontal eccentricity are plotted for both the left and right eyes of all CHM participants (top two rows) and HCs (bottom two rows). Across all plots, visual sensitivity (dB) is plotted on the y-axis, with higher sensitivity reflecting better vision. Across all plots, horizontal position (deg) is plotted on the x-axis, with the Nasal (N) and Temporal (T) visual fields labeled. For all plots, the gray-box denotes sensitivity values between 30-45 dB, which reflects high sensitivity/good vision. Eccentricity profiles in CHM participants are plotted in the top two rows. P1 demonstrates largely normal vision, with the majority of positions falling within the gray-box. In contrast, P2 and P3 exhibit eccentricity profiles that only overlap with the gray-box at largely central locations. P4 exhibits a striking eccentricity profile, with a pronounced peak in sensitivity at the center of the visual field in both eyes, which does not overlap the gray-box. Eccentricity profiles in HCs are plotted in the bottom two rows. All, HCs exhibit eccentricity profiles that are largely flat and overlap with the gray-box across the entire extent of the tested visual field.

Quantification of Relationship Between Clinical and pRF Horizontal Eccentricity Profiles

To show the high predictability of the pRF measures for CHM participants' eccentricity profiles, the visual field coverage maps needed to be converted to represent a similar spatial distribution as the clinically measured eccentricity profiles (Supplementary Figure 5). In accordance with the clinical assessment of eccentricity, pRF maps were restricted to include only the first 4 degrees of visual angle in the vertical dimension (± 2 degrees superior and inferior to the horizontal meridian) across the entire width of the visual field (± 10 degrees eccentricity) (Supplementary Figure 5A, 5B). This restricted field of view was then divided into 11 vertical vectors spanning evenly the entire width of the resampled visual field, to match the spatial sampling of clinical eccentricity assessments. The average pRF value in each vertical vector was then calculated and plotted as a function of horizontal location (Supplementary Figure 5D).

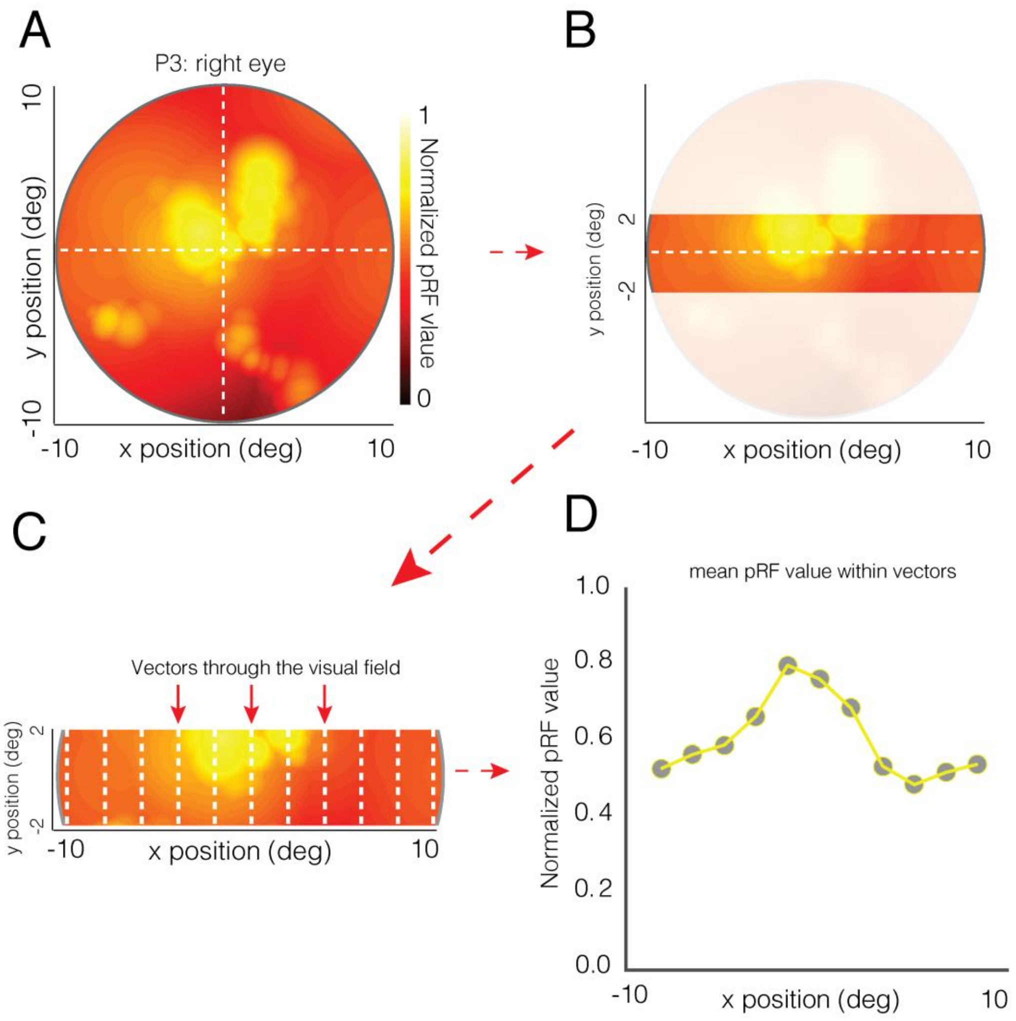

Supplementary Figure 5. Schematic for establishing pRF measurement that resembles the measurement for the clinically assessed horizontal eccentricity sensitivity. **(A)** An example is shown using the visual field coverage for the right eye of P3, normalized to the maximum value in HC1. Hot colors represent regions of the visual field with greater pRF coverage and darker regions represent weaker pRF coverage. **(B)** Following the method of acquisition for the clinical assessment of sensitivity of horizontal eccentricity, the visual field coverage matrix was limited to a restricted field of ± 2 degrees along the y-dimension and ± 10 degrees along the x-dimension. **(C)** Similar to the clinical eccentricity measurements, pRF values along the width of the visual field were divided into 11 linearly spaced vertical vectors and the average pRF values along each vector were calculated. **(D) T**he mean pRF value as a function of horizontal eccentricity for P3 is plotted, representing the strength of pRF responses at different horizontal positions in the visual field.

Eccentricity Measurements Across the Cortical Surface

Although the majority of analyses were conducted on the significantly modulated voxels pooled across both hemispheres, it is also noteworthy to demonstrate the distribution of pRF eccentricity across the cortical surface. The cortical representation of eccentricity, derived from the right eye pRF scans, are shown on surface reconstructions of both left and right hemispheres for a representative HC (HC1), and two CHM patients at their early and late disease stages respectively (P1 and P4), (see Supplementary Figure 6). As expected, the cortical distribution of eccentricity in HC1 follows a well described and predictable pattern[16] for a typical healthy sighted control, extending gradually from the occipital pole, representing central vision, to representations of the peripheral visual field more anteriorly (top row Supplementary Figure 6). Similar to HC1, the cortical distribution of eccentricity in P1 (early stage) shows a general posterior to anterior progression from foveal to peripheral, although the eccentricity distribution in P1 appears to be shifted posteriorly relative to HC1 (middle row Supplementary Figure 6). In stark contrast to both HC1 and P1, the cortical distribution of eccentricity in P4 (late stage) is almost exclusively foveal and is shifted anteriorly (bottom row Supplementary Figure 6).

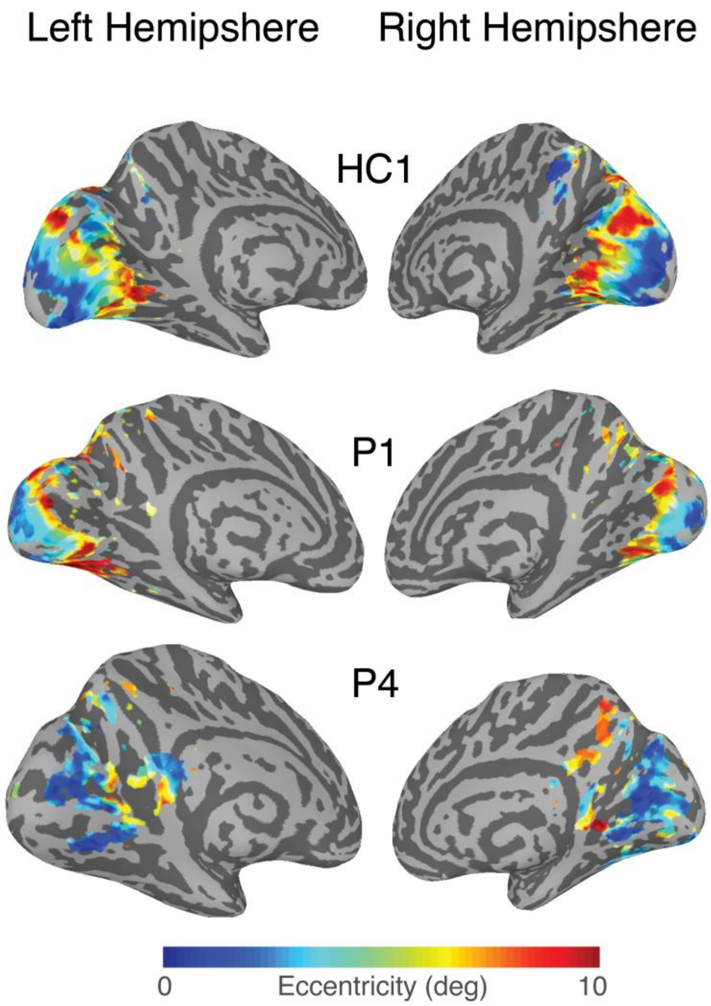

Supplementary Figure 6. Right eye pRF eccentricity distribution depicted on medial aspects of the left and right inflated hemispheres (dark and light gray colors represent sulci and gyri respectively) for a healthy control (HC1) and two CHM patients, P1 and P4 who are at early and late disease stages respectively. To best demonstrate differences between the HC1 and CHM patients, spatial distribution of eccentricity from fovea (Central position = 0) to the peripheral visual field (peripheral location = 10 degrees) are overlaid using false color where dark blue represents foveal vision, red represents the peripheral visual field at 10 degrees and light blue, yellow and green represents the visual spaces between 0-10 degrees. As anticipated, the representation of eccentricity in HC1 progresses gradually from the fovea (dark blue) at the occipital pole, towards the representation of the periphery (red) more anteriorly. The spatial distribution of eccentricity in P1 with early stage disease also shows a general progression from foveal posteriorly towards more peripheral anteriorly, although relative to HC1, the visual field is

primarily limited to central visual field (blue colors) with a more restricted peripheral representation that is considerably shifted posteriorly. In addition, there is far less representation of the periphery dorsally than ventrally. In contrast to both HC1 and P1, the spatial distribution of eccentricity for P4 is strikingly different. Almost all of the cortical representation of the visual field is limited to foveal vision (blue) with an overall anterior shift

Alternative pRF Implementations

Although all pRF models follow a similar general approach, some differences exist. For instance, the pRF implementation used in the current study applies a linear model, similar to the original pRF description[20], but more recent pRF implementations include compressive spatial nonlinearities[22] and deviations from strictly circular pRF shapes[21]. Data analyzed with these different implementations may produce subtlety different pRF estimates, although it is unlikely that these subtle differences would change the overall pattern of results reported here, namely the strong positive relationship reported between the pRF and clinically measured data for CHM participants. Nevertheless, future studies could test the effect that different pRF implementations have on the correspondence between clinical and pRF measurements.

It is also worth noting that although CHM participants and controls performed a task at the fixation point, they were not performing a task on the stimulus explicitly. A number of previous studies have investigated the effect of stimulus task on fMRI signals in visual cortex of patients with retinitis pigmentosa[28] and macular degeneration[42], respectively. It is possible that performing a task on the stimulus[43] may alter the patterns of activity in the periphery of participants with CHM and future studies could explore this possibility.

Reliability of pRF estimates

Unfortunately, in the current CHM and HC groups only a single ~3-minute pRF run was acquired for each eye separately, which makes quantification of pRF reliability difficult. Ideally multiple runs for each eye would be acquired in order to demonstrate the reliability and

robustness of the pRF estimates that this model derives. However, previous work[31] employing the exact computational model used here, has reported the reliability of the pRF estimates across independent sets of data in healthy participants. This report, demonstrates high degree of reliability ($0.68 < r < 0.94$) across the three main parameters of interest and provides reasonable confidence with respect to the pRF measurements acquired in the current experiment.
